# Positron emission tomography in the diagnosis and follow-up of transthyretin amyloid cardiomyopathy patients: A systematic review

**DOI:** 10.1007/s00259-023-06381-3

**Published:** 2023-08-10

**Authors:** H. S. A. Tingen, A. Tubben, J. H. van ’t Oever, E. M. Pastoor, P. P. A. van Zon, H. L. A. Nienhuis, P. van der Meer, R. H J. A. Slart

**Affiliations:** 1 Department of Nuclear Medicine and Molecular Imaging, University Medical Center Groningen, Hanzeplein 1, 9713GZ, Groningen, The Netherlands; 2 Amyloidosis Centre of Expertise, University Medical Center Groningen, Hanzeplein 1, 9713GZ, Groningen, The Netherlands; 3 Department of Cardiology, University Medical Center Groningen, Hanzeplein 1, 9713GZ, Groningen, The Netherlands; 4 Department of Internal Medicine, University Medical Center Groningen, Hanzeplein 1, 9713GZ, Groningen, The Netherlands; 5 Biomedical Photonic Imaging Group, Faculty of Science and Technology, University of Twente, Enschede, The Netherlands

**Keywords:** ATTR, cardiomyopathy, PET, diagnostic accuracy, monitoring therapy

## Abstract

**Purpose:**

Transthyretin (ATTR) amyloidosis is a progressive protein misfolding disease with frequent cardiac involvement. This review aims to determine the value of PET in diagnosis, assessment of disease progression or treatment response and its relation to clinical outcome in follow-up of ATTR amyloid cardiomyopathy (ATTR-CM) patients.

**Methods:**

Medline, Cochrane Library, Embase and Web of Science databases were searched, from the earliest date available until December 2022, for studies investigating the use of PET in ATTR-CM patients. Studies containing original data were included, except for case reports. Risk of bias was assessed by QUADAS-2.

**Results:**

Twenty-one studies were included in this systematic review, investigating five different tracers: carbon-11 Pittsburgh compound B ([^11^C]PIB), fluorine-18 Florbetaben ([^18^F]FBB), fluorine-18 Florbetapir ([^18^F]FBP), fluorine-18 Flutemetamol ([^18^F]FMM) and fluorine-18 Sodium Fluoride (Na[^18^F]F). In total 211 ATTR amyloidosis patients were included. A majority of studies concluded that [^11^C]PIB, [^18^F]FBP and Na[^18^F]F can distinguish ATTR amyloidosis patients from controls, and that [^11^C]PIB and Na[^18^F]F, but not [^18^F]FBP, can distinguish ATTR-CM patients from patients with cardiac light chain amyloidosis. Evidence on the performance of [^18^F]FBB and [^18^F]FMM was contradictory. No studies on the use of PET in follow-up were found.

**Conclusion:**

[^11^C]PIB, Na[^18^F]F and [^18^F]FBP can be used to diagnose cardiac amyloidosis, although [^18^F]FBP may not be suitable for the distinction of different types of amyloid cardiomyopathy. No studies on PET in the follow-up of ATTR amyloidosis patients were found. Future research should focus on the use of these PET tracers in the follow-up of ATTR amyloidosis patients.

**Supplementary Information:**

The online version contains supplementary material available at 10.1007/s00259-023-06381-3.

## Introduction

Systemic amyloidosis is a group of rare and progressive protein misfolding diseases characterized by extracellular deposition of insoluble amyloid fibrils in a variety of tissues [1, 2]. Amyloid deposits interrupt normal tissue structure and induce organ dysfunction [3]. In transthyretin (ATTR) amyloidosis, amyloid is derived from the protein transthyretin and deposits frequently occur in the heart, leading to ATTR cardiomyopathy (ATTR-CM) [2, 4]. The presence and severity of cardiomyopathy are important predictors of morbidity and mortality [5] and its progression should therefore be closely monitored.

ATTR-CM is currently diagnosed and monitored by a combination of presence and severity of symptoms, cardiac biomarkers, electrocardiography, tissue biopsy and several imaging techniques, including echocardiography, cardiac magnetic resonance imaging and bone scintigraphy [4, 6]. Addition of positron emission tomography (PET) might improve diagnosis and monitoring of ATTR-CM patients and is one of the suggested topics of future research in the ESC position statement paper [4]. PET has a good spatial resolution, potentially allowing for earlier diagnosis and more accurate follow-up of patients to evaluate therapy effect. Additionally, quantification of cardiac tracer uptake on PET images could allow for more accurate detection of treatment response or disease progression and could potentially predict clinical outcomes. In the near future, when multiple treatment options might be available for individual patients [7], this will allow clinicians to confidently make treatment related decisions to optimize therapy on an individual level.

Multiple PET tracers have already been studied for diagnosing ATTR-CM [8], however, PET has not yet been implemented in the diagnostic work-up of ATTR-CM and its ability to detect disease progression or treatment response remains unclear.

The primary objective of this review is to determine which tracers have the highest diagnostic accuracy for diagnosing ATTR-CM. The secondary objective is to identify the most promising tracer(s) for future research with regard to the use of PET to detect treatment response or disease progression in the follow-up of ATTR-CM patients.

## Methods

### Data sources and search strategy

This review has been conducted according to previously published guidelines [9] and has been reported according to the PRISMA diagnostic test accuracy guidelines [10]. The review protocol can be found in the PROSPERO database (CRD42022352748).

Medline, the Cochrane library, Embase and Web of Science were comprehensively searched from the earliest available date to December 2022. No language restrictions were applied. Animal studies and case reports were excluded. The search string included medical subject headings and free text and consisted of disease related terms; “amyloidosis”, “ATTR”, “transthyretin”, and “cardiac amyloidosis” and exposure related terms; “Positron-Emission Tomography”, and “PET/CT”. The full search strategy per database is displayed in online resource 1.

After study selection, reference lists of previously conducted systematic reviews on a similar topic and reference lists of all included studies were searched for additional references.

### Study selection

Studies were included if original data were presented and if the diagnostic value of PET or the value of PET in the follow-up of ATTR-CM patients to detect treatment response or disease progression was discussed. No additional exclusion criteria were applied.

All studies were collected in Rayyan (https://www.rayyan.ai) and duplicates were eliminated. Two researchers (PZ, EO) independently screened and critically assessed the studies for relevance, based on title and abstract. Disagreements were resolved by a third and fourth independent researcher (AT, HT). Full texts were retrieved and assessed by two independent researchers for relevance (PZ, EO). A third and fourth independent (AT, HT) researcher were available to resolve disagreement. If full text article were not retrievable, authors were contacted to ascertain that no full text article were published or obtainable.

### Data extraction and quality assessment

One researcher (EP) extracted relevant data from the included studies using a previously made extraction form and extraction was checked by a second reviewer (EO), disagreements were settled by a third and fourth reviewer (AT, HT). A list of all items included in the data extraction form is provided in online resource 2. The quality of the included studies was assessed by two independent researchers (AT, HT) using the Quality Assessment of Diagnostic Accuracy Studies-2 (QUADAS-2) [11]. The QUADAS-2 was used to assess the risk of bias of the included articles with regard to patient selection, index test, reference test and flow/timing, and applicability concerns were assessed for patient selection, index and reference test. Additionally, included studies were assessed for potential overlap of patient groups.

### Data synthesis and statistical analysis

The interobserver agreement for article inclusion was assessed by calculating Cohen’s kappa. Outcomes of the included studies were summarized and compared. To facilitate the comparison, subgroups were formed based on the studied PET tracer and whether data about diagnostic accuracy or value in follow-up were gathered. Outcomes of studies are represented as mean ± standard deviation or as median [interquartile range] where applicable. Outcomes of interest regarding diagnostic accuracy were sensitivity, specificity or accuracy to detect ATTR-CM patients by PET from any population or differences in cardiac tracer uptake (in any presented unit) between ATTR-CM patients and any control group. Follow-up outcomes of interest were the noted difference in cardiac tracer uptake between sequential scans (in any presented unit), if available in relation to other imaging modalities or clinical parameters in treated or untreated patients.

## Results

### Literature search and selection of studies

**Fig. 1:**
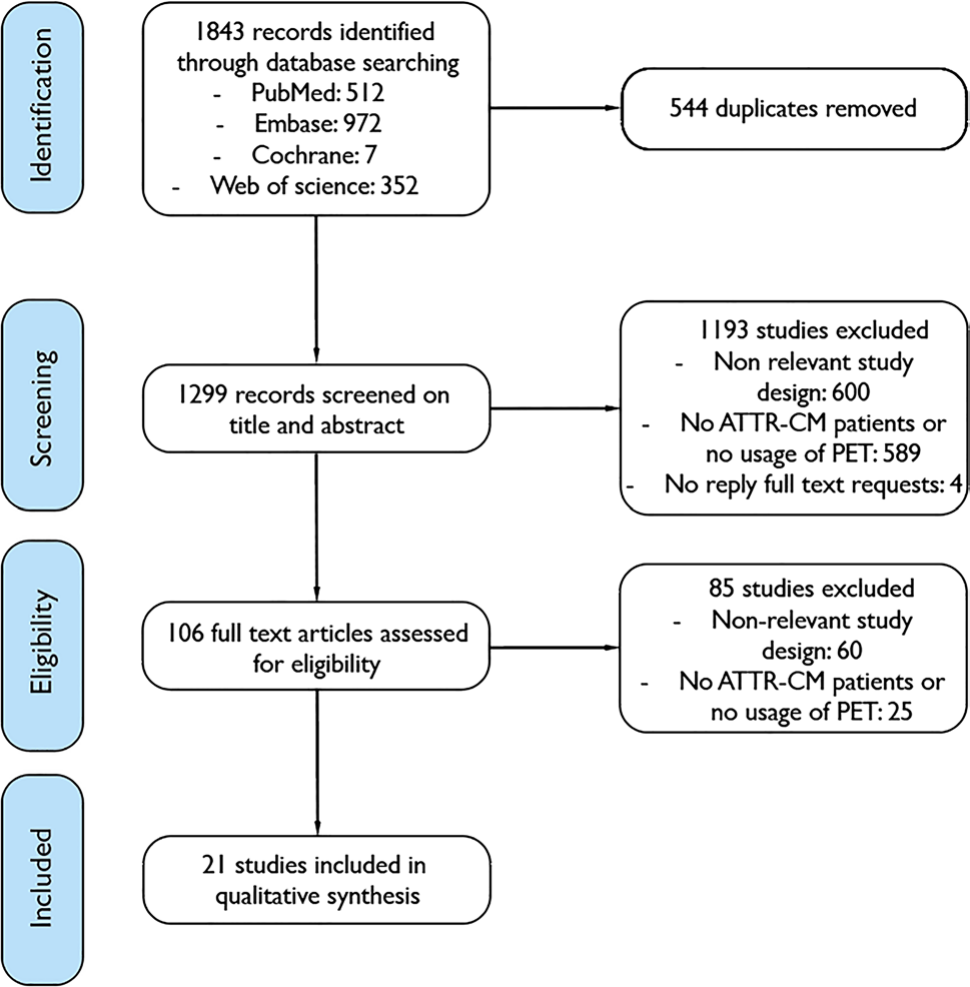
Flow chart of inclusion of studies

The search resulted in 1843 studies, of which 544 duplicates. A total of 1193 non-relevant studies, 600 studies with a non-relevant study design and 589 studies without ATTR-CM patients or without the use of PET were excluded based on abstract and title. Of four studies, no answer to full text requests was provided and no further subclassification could be provided. One hundred six full text articles were screened by two reviewers with perfect agreement (Cohen’s kappa 0.898; PZ, EO). Twenty one studies were included [12–32]. Of the 85 excluded full-text studies, 60 did not have a relevant study design and 25 studies did either not investigate PET or did not include ATTR-CM patients. The inclusion process is shown in a flow chart in Fig. 1.

### Quality assessment

**Fig. 2:**
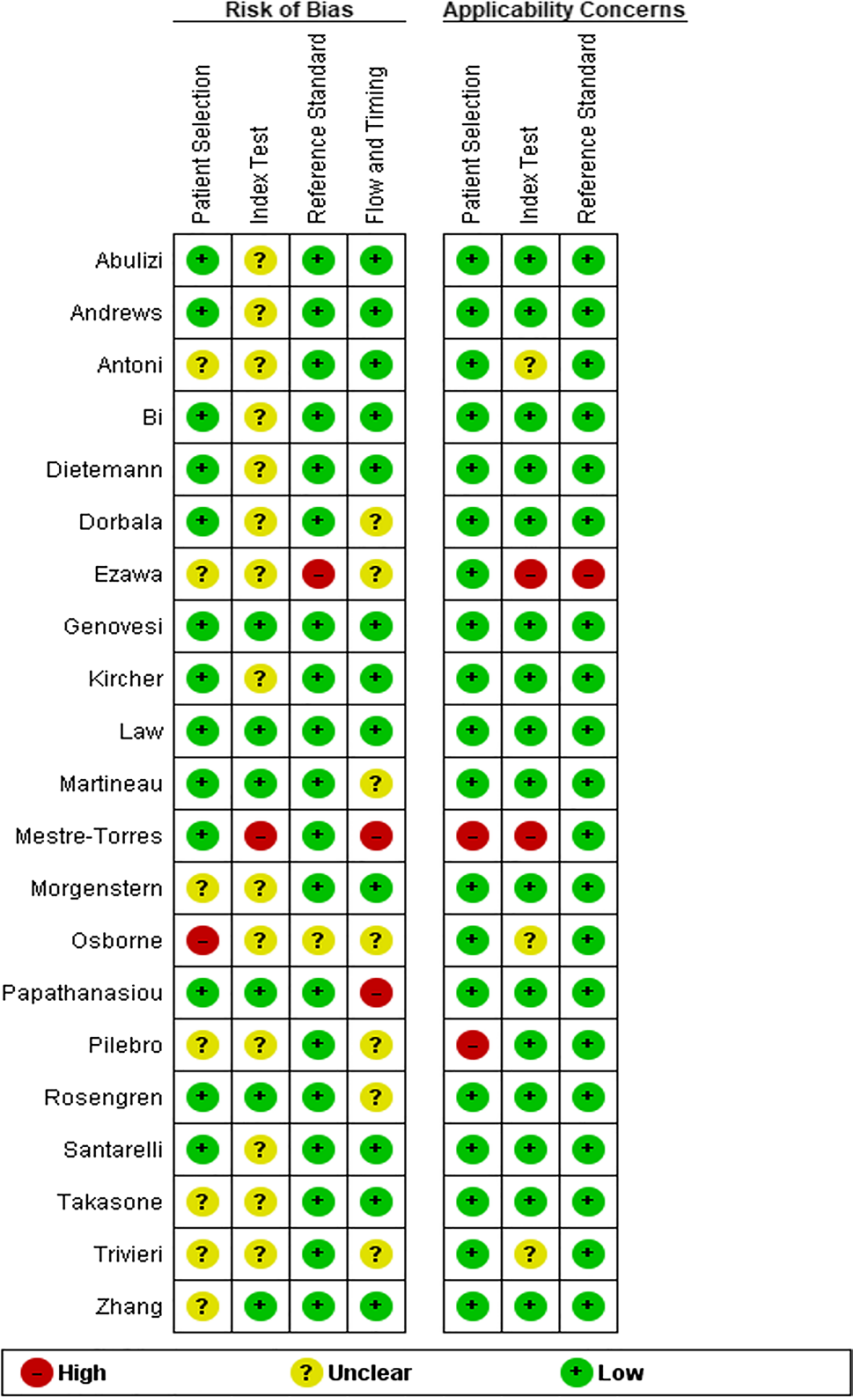
Risk of bias and applicability concerns summary according to QUADAS-2

The quality of the included studies was assessed using the QUADAS-2 [11] and is shown in Fig. 2. The overall quality of the included studies was satisfactory. There was a low risk of overlapping study populations in studies investigating fluorine-18 Florbetapir ([^18^F]FBP) and fluorine-18 Flutemetamol ([^18^F]FMM), and unclear risk in studies investigating fluorine-18 florbetaben ([^18^F]FBB) and fluorine-18 Sodium Fluoride (Na[^18^F]F) and high risk in two studies investigating carbon-11 Pittsburgh Compound B ([^11^C]PIB). A substantiation of the risk of overlap can be found in online resource 3.

### Clinical characteristics

**Fig. 3:**
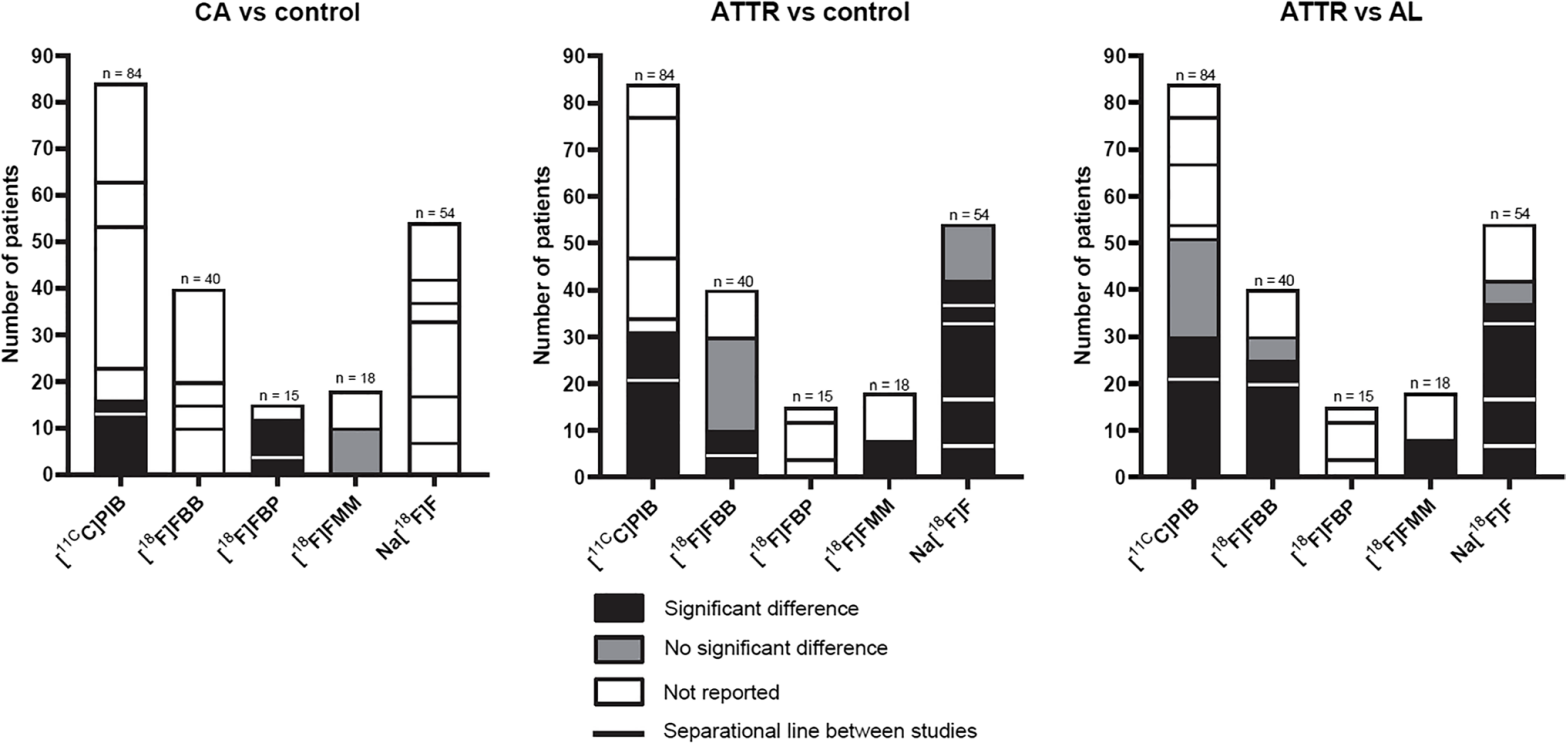
Reported outcomes per study per tracer for the comparison between different study groups. n = total number of included patients per tracer

**Fig. 4:**
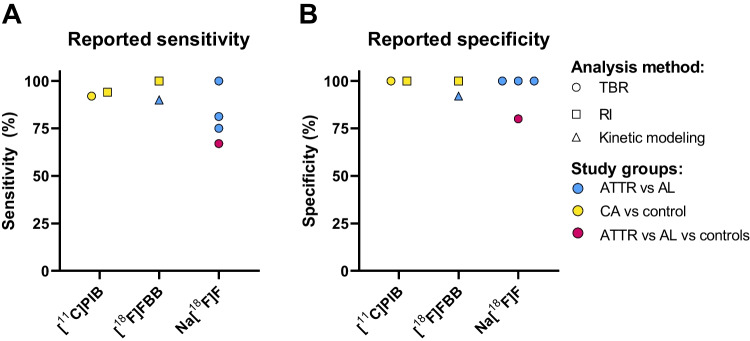
Reported accuracy outcomes per study per tracer for the comparison between different study groups; **A**. Reported sensitivity; **B**. Reported specificity

Of the 21 included studies, six investigated [^11^C]PIB, four investigated [^18^F]FBB, three investigated [^18^F]FBP, two investigated [^18^F]FMM and six investigated Na[^18^F]F. A total of 211 ATTR amyloidosis patients were included. Some studies included both ATTR and light chain (AL) amyloidosis patients and did not distinguish between these subtypes of amyloidosis in their results section, while other studies included ATTR amyloidosis patients only or presented AL and ATTR amyloidosis patients as subgroups in their results section. Two of the included studies did not report general clinical characteristics of their study group, including mean age and percentage of male patients [23, 25]. The majority of the included studies were of prospective design (67%). Additional characteristics of all studies are shown in Table 1. All studies compared the performance of PET to the clinical diagnosis. More details on how clinical diagnosis was made is provided in online resource 4. A graphical overview of the outcomes of all studies is provided in Figs 3 and 4.

**Table 1:** Study characteristics of the included studies

General characteristics				Study group characteristics			Control group characteristics	
Authors, year (reference no.)	Study design	Outcome	Tracer	n	Amyloidosis type	Age	n	Condition(s)
[^11^C]PIB								
Antoni, 2012 [12]	R	D	[^11^C]PIB	10	ATTR = 1 ATTRv = 2 AL = 7		5	Healthy
Bi, 2022 [13]	R	D	[^11^C]PIB	13	AL + ATTR	62.9 ± 8.2	18	DCM, RHD, VHD, HHD, HCM, healthy
Rosengren, 2020 [14]	P	D	[^11^C]PIB	21	ATTRv = 5 ATTRwt = 16	76 (12.5)	30	AL, non-amyloid cardiac hypertrophy, healthy
Pilebro, 2016 [15]	P	D	[^11^C]PIB	10	ATTRv-CM: 7 NP ATTRv-CM: 3	68.7 ± 3.9	5	Healthy
Takasone, 2020 [16]	P	D	[^11^C]PIB	30	ATTRv = 22 ATTRwt = 8	57.8 ± 19.5	17	AL
Ezawa, 2018 [17]	P	D	[^11^C]PIB	7	ATTRv = 7	44.3 ± 11.3	11	AL, healthy
[^18^F]FBB								
Genovesi, 2021 [18]	P	D	[^18^F]FBB	20	ATTRwt = 20	80.7 ± 7.2	40	AL, HCM, HHD, DCM
Kircher, 2019 [19]	R	D + F	[^18^F]FBB	5	ATTR = 2 ATTRwt = 3	73.2 ± 5.36	17	AL, AA, LCDD, Systemic AL, Cutaneous AL, MM AL, CAD, MM systemic AL, FMF AA, HCM
Law, 2016 [20]	P	D	[^18^F]FBB	5	ATTRwt = 5	72.6 ± 9.2	9	AL, HHD
Santarelli. 2022 [21]	R	D	[^18^F]FBB	10	ATTR = 10	82 ± 8	26	AL, HHD, HCM, AoS
[^18^F]FBP								
Dorbala, 2014 [22]	P	D	[^18^F]FBP	4	ATTR = 4	73 ± 5.23	10	AL, Healthy, NICMP
Mestre-Torres, 2018 [24]	P	D	[^18^F]FBP	3	ATTRv = 2 ATTRwt = 1	70 (61.5 -75.5)	22	AL, AA, Alzheimer
Osborne, 2015 [23]	P	D	[^18^F]FBP	8	ATTR = 4 ATTRwt = 4	NR	3	Healthy
[^18^F]FMM								
Dietemann, 2019 [25]	R	D	[^18^F]FMM	9	ATTR = 8 AL = 1	NR	3	Healthy
Papathanasiou, 2020 [26]	R	D	[^18^F]FMM	12	ATTRv = 3 ATTRwt = 7 AL = 2	71.3 ± 9.5	5	Non-amyloid HF
Na[^18^F]F								
Andrews, 2020 [28]	P	D	Na[^18^F]F	10	ATTR = 10	70 ± 9	43	AL, healthy, aortic stenosis
Abulizi, 2019 [29]	P	D	Na[^18^F]F	16	ATTRv = 9 ATTRwt = 7	73 ± 9	11	AL
Trivieri, 2016 [30]	P	D	Na[^18^F]F	4	ATTRv = 2 ATTRwt = 2	67 ± 8	10	AL, healthy
Martineau, 2019 [27]	R	D	Na[^18^F]F	7	ATTR = 7	76.43 ± 6.6	8	AL, HCM, ICMP
Morgenstern, 2017 [31]	P	D	Na[^18^F]F	5	ATTRv = 2 ATTRwt = 3	66.6 ± 8.44	7	AL, prostate cancer
Zhang, 2020 [32]	P	D	Na[^18^F]F	12	ATTR = 11 ATTRv = 1	79 ± 8	5	Healthy

No., number; n, number of patients; NR, not reported. Study design: P, prospective; R, retrospective; C, case report. Amyloidosis type: ATTR, transthyretin amyloidosis subtype unspecified; ATTRv, hereditary transthyretin amyloidosis; ATTRwt, wild-type transthyretin amyloidosis; Not proven. Condition(s): AA, AA amyloidosis; AL, light-chain amyloidosis; AoS, aortic valve stenosis; CAD, coronary artery disease; DCM, dilated cardiomyopathy; FMF, familial Mediterranean fever; HCM, hypertrophic cardiomyopathy; HF, heart failure; HHD, hypertensive heart disease; ICMP, ischemic cardiomyopathy; LCDD, light-chain deposition disease; MM, multiple myeloma; CTS-, smoldering myeloma, Non-CA ATTR; RHD, rheumatic heart disease; VHD, valvular heart disease; NICMP, non-ischemic cardiomyopathy. Tracers: Na[^18^F]F, fluorine-18 Sodium Fluoride; [^11^C]PIB, carbon-11 Pittsburgh Compound B; [^18^F]FMM, fluorine-18 Flutemetamol; [^18^F]FBP, fluorine-18 Florbetapir; [^18^F]FBB, fluorine-18 florbetaben. Acquisition: S, start time; D, duration. Outcome: D, diagnostic; F, follow-up. Assessment; AUC, area under the curve; MTR, myocardial tracer retention; MV, molecular volume; NPV, negative predictive value; PPV, positive predictive value; RI, retention index; ROC, receiver operating characteristics; SUV, standardized uptake value; TBR, tissue-to-background ratio; VI, visual interpretation

### PET characteristics and performance in the diagnosis of ATTR-CM

The main characteristics and results of the included studies will be summarized below. A more detailed summary of the technical aspects and results can be found in Table 2.

**Table 2:** PET characteristics and results of the included studies

Authors, year (reference no.)	Acquisition			Results				
	Tracer dose	Dynamic (min)	Static (min)	*RI*	*TBR*	*SUV*	*Accuracy*	*Other*
[^11^C]PIB; ordered based on start static scan								
Antoni, 2012 [12]	10 MBq/kg	D: 32, 25 S: 0, 120		RI_mean_: • CA: 0.054 (0.033 - 0.134) • HC: 0.025 (0.020 - 0.031), (p=.0007)				
Rosengren, 2020 [14]	5MBq/kg	D: 35 S: 0	D: 10 S: 10	10-20 minutes RI: • ATTR: 0.045 min^-1^ [0.014] • AL: 0.086 min^-1^ [0.075] • HCM: 0.029 min^-1^ [0.005], (p<.001) • HC: 0.033 min^-1^ [0.005], (p<.001)	10-20 minutes TBR_mean_: • ATTR: 1.64 [0.62] • AL: 2.61 [2.61] • HCM: 0.88 [0.26] • HC: 0.87 [0.26], (p<.001)		Cutoff TBR: 1.09 CA vs controls • AUC: 0.98 CI: 0.94-1.00) • Sen: 94% (CI: 80-99%) • Spec: 93% (CI: 66-100%) Cutoff RI: 0.037 CA vs controls • Accuracy: 96% (CI: 87-100%) • Sen: 94% (CI: 80-99%) • Spec: 100% (CI: 75-100%)	
Pilebro, 2018 [15]	6MBq/kg	D: 25 S: 0	D: 10 S: 15	RI: • ATTRv: 0.084 [0.032 - 0.179] • HC: 0.025 [0.020 – 0.031], (p<.001)				
Ezawa, 2018 [17]	500-636 MBq		D: 14 S: 30					VI: ATTRv: +++: 3 ++: 2 +:1 -: 1 • AL: +++: 2 ++: 2 +: 2 -: 1; • Healthy control: -: 3.
Takasone, 2020 [16]	500-636 MBq		D: 14 S: 30			SUV_max_: • ATTRwt: 1.42 [0.38 – 1.92] • Early onset ATTRv V30M: 5.64 [3.47 – 8.18], (p=.0019) • Late onset ATTRv V30M: 1.65 [1.21 – 2.12] • ATTRv other: 2.01 [1.5 – 2.19] • AL: 6.16 [2.14 – 19.2], p = .0002.		
Bi, 2022 [13]	555 MBq		D: 20 S: 30		TBR_max_: • CA: 2.66 ± 1.99 • Non-CA HF: 0.85 ± 0.06, (p<.05) • HC: 0.88 ± 0.07, (p=.003)		Cut-off TBR: 1.09 CA vs controls • AUC: 0.99 (CI: 0.96-1.00) • Sens: 92% (CI: 62-100%) • Spec: 100% (CI: 78-100%)	
[^18^F]FBB; ordered based on start static scan								
Santarelli, 2022 [21]	300 MBq	D: 40 S:0					Kinetic modelling: • Sen: 90% • Spec: 92% • Accuracy: 97%	
Kircher, 2019 [19]	313 ± 26 MBq	D: 30 S: 0	D: 20 S: 10	MTR: • ATTR: 42 (38-45) • AL: 66 (38-11) (p<.01) • AA: 58 • Non-CA: 27 (21-34)			RI cut-off: ≤ 36 CA vs non-CA • AUC 1.0 • Sen: 100% • Spec: 100% (p <.001) ≤ 52 ATTR vs AL+ AA • AUC: 0.91% • Sen: 100% Spec: 89% (p <.005)	
Law, 2016 [20]	259 ± 41 MBq	D: 80 S: 0	D: 60 S: 15	RI: • ATTR: 0.035 [0.022 - 0.042] min^-1^ • AL: 0.043 [0.032 - 0.065] min^-1^ • Hypertensive controls: 0.010 min^-1^ [0.008 - 0.015] MTR • ATTR: 71.2% (51.3% - 104.7%) • AL: 76.2% [45.3%-157.2%], (p>0.99) • Hypertensive controls: 28.8% [24.5-35.4%], (p=.042)				
Genovesi, 2021 [18]	300 MBq	D: 60 S: 0	D: 10 S: 5, 30, 50, 110		50-60min TBR_mean_: • ATTR: 1.73 [1.30-1.82] • AL: 4.27 [2.45-5.05], (p<.001) • Non-CA: 1.55 [1.44-1.80], (p=.987)	50-60min SUV_mean_: • ATTR: 1.45 [1.15-1.80] • AL: 4.70 [3.60-6.91], (p=.001) • Non-CA: 1.60 [1.27-1.87], (p=.552)		50-60min MV: • ATTR: 52.0 [39.0-108.0]; • AL: 200.0 [170.0-238.2], (p<.001) Non-CA: 14.0 [9.50-16.9], (p<.001)
[^18^F]FBP; ordered based on start static scan								
Osborne, 2015 [23]	370 MBq	D: 30 S: 0	D: 3, 5, 5 S: 0, 10, 15		SUV_mean-ratio_ 3:15min_:_ • CA: 2.3 ± 1.4 HC: 6.2 ± 0.95	SUV_mean_ 15min_:_ • CA: 4.7 ± 1.7 • HC: 1.4 ± 0.2		Uptake rate of change of SUV 0-20 minutes: • CA: 0.28 SUV/min • HC: 5.4 SUV/min, (p<.01)
Dorbala, 2014 [22]	222 MBq	D: 60 S: 0	D: 50 S: 10	RI: CA: 0.043 [0.034-0.051] min^-1^ Control: 0.023 [0.015-0.024] min^-1^, (p=.002)	TBR_mean_: • CA: 1.84 [1.64-2.50] • Control: 1.26 [0.91-1.36], (p=.001)	SUV_mean_: • CA: 3.84 [1.87-5.65] • Control: 1.35 [1.17-2.28], (p=.03)		
Mestre-Torres, 2018 [24]	370 MBq		D: 10 S: 40		TBR_mean_: • CA: 5.12 ± 4.95 • CA-negative: 2.94 ± 1.32 • Control: 2.39 ± 0.78			
[^18^F]FMM; ordered based on start static scan								
Dietemann, 2019 [25]	360 MBq	D: 30 S: 0	D: 20 S: 10		TBR_mean_: • ATTR: 1.44 [1.33-1.69] • AL: 3.0 • Control: 1.06 [0.72-1.1], (p=.033)			
Papathanasiou, 2020 [26]	182.1 ± 18.5 MBq	D: 30 S: 0	D: 30 S: 60		TBR_mean_: • CA: 1.4 ± 0.8 • Non-CA HF: 0.9 ± 0.1, (p=.11) TBR_max_: • CA: 1.3 ± 0.8 • Non-CA HF: 0.9 ± 0.1, (p=.11)	SUV_mean_: • CA: 1.7 ± 0.8 • Non-CA HF: 1.3 ± 0.3, (p=.13) SUV_max_: • CA: 2.2 ± 1.0 • Non-CA HF: 1.7 ± 0.5, (p=.18)		
Na[^18^F]F; ordered based on start static scan								
Martineau, 2019 [27]	370 MBq	D: 30 S: 60			TBR_mean_: • ATTR: 0.98 ± 0.09 • AL: 0.85 ± 0.08, (p=.026) • HCM/ICMP: 0.82 ± 0.07, (p=.020)	SUV_mean_: • ATTR: 1.31 ± 0.30 • AL: 0.84 ± 0.55, (p>.05) • Controls: 1.29 ± 0.21, (p>0.05)	VI: ATTR vs AL + controls • Sens: 57% (CI: 18-90%) • Spec: 100% (CI: 63-100%) Cut-off TBR_mean_ 0.89 ATTR vs AL + controls • AUC 0.91 ± 0.08 • Sen:75% (CI: 35-97) • Spec: 100% (CI:59-100%), (p=.0078)	
Morgenstern, 2017 [31]	370 ± 1.2 MBq		D: 10 S: 60 (1x 90)			SUV_mean_: • ATTR: 1.5 (1.4-1.7) (ATTRwt 1.7 (1.4-1.8), ATTRv 1.45 (1.4-1.5)) • AL: 0.95 (0.9-1.0), (p=.078) Control: 0.8 (0.4-0.9), (p=.012)		
Abulizi, 2019 [29]	4 MBq/kg		D: 60 S: 60		TBR_mean_: • ATTR: 1.00 ± 0.12 (ATTRwt 1.01 ± 0.13, ATTRv 0.98 ± 0.12) • AL 0.81 ± 0.06, (p=.001) • No CA: 0.73 ± 0.16, (p=.006)		Cutoff TBR_mean_ ≥ 0.90 ATTR vs AL • AUC: 0.937 • Sen: 81.2% • Spec: 100%, (p=.0005)	
Andrews, 2020 [28]	125-350 MBq		D: 60 S: 60		TBR_mean_: • ATTR: 1.13 ± 0.16 • AL: 0.95 ± 0.08, (p=.01) • AoS: 0.73 ± 0.12, (p<.0001) HC: 0.86 ± 0.10, (p=.0002)		Cut-off TBR_mean_ > 1.14 ATTR vs AL after LGE linking • AUC: 1.00 • Sen: 100% (CI:72.25-100%) Spec: 100% (CI: 67,56-100%), (p=.0004)	
Trivieri, 2016 [30]	386 ± 69 MBq	D: 90 S: 5	D: 30 S: 65		TBR_max:_ • ATTR: 1.14 ± 0.24 • AL: 0.77 ± 0.66, (p<.05) • Control: 0.68 ± 0.04, (p=.001)			
Zhang, 2020 [32]	370 MBq	D: 30 S: 60	D: 15 S: 180		TBR_mean_ 1h: • ATTR: 0.83 ± 0.15 • Controls: 0.72 ± 0.15, p=.23 TBR_mean_ 3h: • ATTR: 0.88 ± 0.26 • Controls: 0.70 ± 0.04 (p=.20)		VI 1 hour • Sen: 25% (CI: 8.9-53%) • Spec: 100% (CI: 57-100%) • Accuracy: 47% VI 3 hours • Sen: 30% (11-60%) • Spec: 100% Cut-off TBR_mean_ 1 hour >0.76, ATTR-CM vs controls • AUC: 0,69 (CI: 0.37-1.00) • Sen: 67% (CI: 39-86%) • Spec: 80% (CI: 38-99%)	

Diseases: ATTR, transthyretin amyloidosis subtype unspecified; ATTRv, hereditary transthyretin amyloidosis; ATTRwt, wild-type transthyretin amyloidosis; CA, cardiac amyloidosis; HF, heart failure; AL, light chain amyloidosis; AA, AA amyloidosis; SM, smoldering myeloma; AoS, aortic stenosis; HCM, hypertrophic cardiomyopathy; ICMP, ischemic cardiomyopathy; CTS, carpal tunnel syndrome: HC, healthy controls. Tracers: Na[^18^F]F, fluorine-18 Sodium Fluoride; [^11^C]PIB, carbon-11 Pittsburgh Compound B; [^18^F]FMM, fluorine-18 Flutemetamol; [^18^F]FBP, fluorine-18 Florbetapir; [^18^F]FBB, fluorine-18 florbetaben. Acquisition: MBq, mega becquerel; S, start time; D, duration. Results: AUC, area under the curve; MTR, myocardial tracer retention; MV, molecular volume; NPV, negative predictive value; PPV, positive predictive value; RI, retention index; SUV, standardized uptake value; TBR, tissue-to-background ratio; VI, visual interpretation; sen, sensitivity; spec, specificity; NR, not reported; CI, interquartile range; median [interquartile range]; median (range); mean ± standard deviation

* all p-values are compared to ATTR, in case of no distinct ATTR group they are compared to cardiac amyloidosis in general

#### [^11^C]PIB

Six studies investigated the use of [^11^C]PIB [12–17] and included a total of 84 patients. Five studies used a PET/CT scanner (83%), while one study used a PET/MR scanner (17%) [13]. The injected dose varied from 5 megabecquerel per kilogram bodyweight (MBq/kg) to 636 megabecquerel (MBq). Dynamic scanning was performed in three studies, with the start of acquisition varying from 0 - 120 minutes post injection and the acquisition time varying from 25 - 32 minutes. Static scanning was performed in three studies, with the start of acquisition varying from 10 - 30 minutes post injection and the scan duration time varying from 10 - 30 minutes. Results were reported using several different methods, including retention index (RI) (50%), maximum tissue-to-background ratio (TBR) (17%), mean TBR (17%), mean standard uptake value (SUV) (17%) and visual interpretation (17%).

Antoni et al. [12] found that the mean retention index (RI) was significantly higher in cardiac amyloidosis (CA) patients (n=10) (0.054 [0.033 - 0.134]) compared to healthy controls (HC) (0.025 [0.020 - 0.031]; p = .0007).   After application of kinetic modelling on this population, SUV and RI discriminated better between subtypes of CA compared with Ki [33]. Bi et al. [13] demonstrated a significant difference (p < .05) in TBR between CA patients (2.66 ± 1.99), non-CA patients (n=13) (0.85 ± 0.06) and HC (0.88 ± 0.07).  Furthermore, a TBR cutoff of 1.09 distinguished CA patients from controls with 92% (95% confidence interval (CI): 62-100%) sensitivity and 100% (CI: 78-100%) specificity. Rosengren et al. [14] found that a TBR cut-off of 1.09 differentiated ATTR amyloidosis patients from hypertrophic cardiomyopathy (HCM) patients and HC with a 94% sensitivity (CI: 80 - 99%) and 93% specificity (CI: 66-100%). Additionally, the TBR in ATTR amyloidosis patients (n=21). (1.64 [0.62]) was significantly lower than that in AL amyloidosis patients (2.61 [2.61]; p < .001) Pilebro et al. [15] found a higher RI in hereditary ATTR (ATTRv) amyloidosis patients (n=10) (0.084 [0.032 – 0.179]) compared to HC (0.025 [0.020 – 0.031]; p < .001). Takasone et al. [16] examined the value of combining PIB-PET imaging and [^99m^Tc]-pyrophosphate scintigraphy in wild type ATTR (ATTRwt), early-onset V30M ATTRv, late-onset V30M ATTRv, non-V30M ATTRv and AL amyloidosis patients (n ATTR=30). The maximum SUV in early-onset V30M ATTRv amyloidosis patients (5.64 [3.47 – 8.18]) and AL amyloidosis patients (6.16 [2.43 – 19.2]) were significantly higher than in ATTRwt amyloidosis patients (1.42 [0.38 – 1.92]; p = .0019 and 0.002), late-onset V30M ATTRv amyloidosis patients (1.65 [1.21 – 2.12]; p = .0201) and non-V30M ATTRv amyloidosis patients (2.01 [1.5 – 2.19]; p = .0061). Ezawa et al.[17] inspected [^11^C]PIB PET images visually and scored myocardial uptake as +++, ++, + or -. Some extent of myocardial tracer uptake was noted in all but one ATTR amyloidosis patients (n=7), all but one AL amyloidosis patient and none of the healthy controls.

#### [^18^F]FBB

In four studies, the diagnostic utility of [^18^F]FBB PET was investigated [18–21]. These studies included a total of 40 patients and all used a PET/CT scanner (100%). Injected tracer dose varied from 259 MBq to 313 MBq. Dynamic scanning was performed in all studies, with the start of acquisition at 0 minutes post injection and the acquisition time varying from 30 – 80 minutes. Static scanning was performed in all studies except for the study by Santarelli et al. [21], with the start of acquisition varying from 5 – 50 minutes post injection and the scan duration time varying from 10 – 30 minutes. Results were reported as mean TBR (25%), mean SUV (25%), TBR (25%), RI (50%) and molecular volume (MV) (25%).

In the study by Genovesi *et al.* [18], the results of an early, intermediate, late and delayed static scan were compared between ATTR amyloidosis (n=20), AL amyloidosis and non-CA patients. Uptake in AL amyloidosis patients remained high after the early scan, but decreased rapidly in ATTR amyloidosis and non-CA patients. It was possible to distinguish ATTR amyloidosis patients from AL amyloidosis patients at all scans based on mean standardized uptake value (SUV_mean_), TBR and molecular volume (MV). ATTR amyloidosis patients could only be distinguished from controls based on MV. Kircher *et al*. [19] determined the optimal TBR cut-off to be 36. This cut-off differentiates CA from non-CA patients with a sensitivity and specificity of 100% (p < .001). A TBR cut-off of 52 was found to differentiate between cardiac amyloidosis due to ATTR amyloidosis (n=5) and serum amyloid A (AA) amyloidosis and AL amyloidosis with a sensitivity of 100% and a specificity of 89% (p < .005). In the study by Law *et al.* [20] ATTR amyloidosis patients (n=5) had a higher median myocardial [^18^F]-FBB retention (71.2% [51.3 -104.7%]) compared to HHD patients (28.8% [24.5 – 35.4%]; p = .042), but no difference was found between ATTR and AL amyloidosis patients (76.2% [45.3 – 157.2%]; p > .99). Santarelli et al. [21] investigated whether ATTR amyloidosis patients (n=10) could be accurately distinguished from patients with suspected CA and AL amyloidosis patients through kinetic model fitting on a dynamic scan. By a two-step model utilizing K_1_, κ_2_ and K_i_, ATTR patients could be distinguished from the other groups with an accuracy of 97%, as CA patients have a combination of a low K_1_ value with a high κ_2_ value, as opposed to control patients who had higher K_1_ and κ_2_ values. Subsequently, a combination of a higher κ_2_ value with a low K_i_ value is characteristic for ATTR amyloidosis patients, while a low κ_2_ value, possibly in combination with a high K_i_ value, is more characteristic of AL amyloidosis patients. Exact cut-offs were not reported.

#### [^18^F]FBP

Three studies investigated the value of [^18^F]FBP PET in diagnosing ATTR-CM patients [22–24], and included a total of 15 patients. All studies used a PET/CT scanner (100%) and the injected dose varied from 222 MBq to 370 MBq. Dynamic scanning was performed in two studies, with the start of acquisition at 0 minutes post injection and the acquisition time varying from 30 - 60 minutes. Static scanning was performed in two studies, with the start of acquisition varying from 0 - 40 minutes post injection and the scan duration time varying from 3 - 10 minutes. Results were reported as RI (33%), mean TBR (67%), mean SUV (67%) and mean standard uptake value ratio (SUVR) (33%).

In the study by Dorbala et al. [22] the myocardial RI was significantly higher in CA patients (0.043 [0.034 - 0.051]) compared to controls (0.023 [0.015 - 0.024]; p = .002). However, no differences in RI were found between AL and ATTR amyloidosis patients (n=4). No differences in TBR, SUV and SUVR were found between any of the groups. In the study by Mestre Torres et al. [24], three ATTR-CM patients were included, but no cardiac tracer uptake was detected on PET for any of these patients. Osborne et al. [23] found that the mean SUV in CA patients (n=8) was significantly higher after 10 and 15 min (10 min: 6.1 ± 1.6; 15 min: 4.7 ± 1.7) in comparison with HC (10 min: 1.7 ± 0.3; 15 min: 1.4 ± 0.2; p < .05), while there was no significant difference in uptake at 3 min post injection.

#### [^18^F]FMM

Two studies investigated the use of [^18^F]FMM in the diagnosis of ATTR-CM and included a total of 18 patients [25, 26]. Both studies used a PET/CT scanner (100%). Injected dose varied from 182.1 to 360 MBq. Dynamic scanning was performed in both studies, with the start of acquisition at 0 minutes post injection and an acquisition time of 30 minutes. Static scanning was performed in both studies as well, with the start of acquisition varying from 10 – 60 minutes post injection and the scan duration time varying from 20 – 30 minutes. Results were reported as mean TBR (100%), maximum TBR (100%), mean SUV (50%), maximum SUV (50%).

Dietemann et al. [25] found that TBR was significantly higher in amyloidosis patients (1.46 [1.32 – 2.06]) compared to controls (1.06 [0.72 – 1.1]; p = .033). In the amyloidosis group, only one AL amyloidosis patient was included and this patient showed a higher TBR (3.0) than the ATTR amyloidosis patients (n=8) (1.44 [1.33 - 1.69]). In the study by Papathanasiou et al. [26] maximum SUV, mean SUV and TBR did not significantly differ between CA (n ATTR=10) and non-amyloid heart failure patients. PET showed a low sensitivity of 16.7% in detecting CA.

#### Na[^18^F]F

In six studies Na[^18^F]F was investigated [27–32] in a total of 54 patients. Three studies used a PET/CT scanner (50%) and three studies used a PET/MR scanner (50%). Injected dose varied from 4 MBq/kg to 386 MBq. Dynamic scanning was performed in three studies (50%), with the start of acquisition varying from 5 - 60 minutes post injection and scan duration time varying from 30 - 90 minutes. Static scanning was performed in five studies (83%), with the start of acquisition varying from 60 - 180 minutes post injection and scan duration time varying from 10 - 60 minutes. Results were reported as mean TBR (67%), maximum TBR (17%) and mean SUV (17%).

Abulizi et al. [29] found a significantly higher TBR in ATTR amyloidosis patients (n=16) (1.00 ± 0.12) compared to AL amyloidosis patients (0.81 ± 0.06; p = .001) and non-CA patients (0.73 ± 0.16; p = .006). The optimal TBR cut-off to discriminate ATTR from AL amyloidosis was 0.90 (sensitivity 81.2%; specificity 100.0%; p = .0005). In the study by Andrews et al. [28] TBR was significantly higher in ATTR amyloidosis patients (n=10) (1.13 ± 0.16) compared to HC (0.86 ± 0.10; p = .0002), patients with aortic stenosis (0.73 ± 0.12; p < .0001) and AL amyloidosis patients (0.95 ± 0.08; p = .01). After linking PET-findings with late gadolinium enhancement on cardiac magnetic resonance imaging, a TBR cutoff og 1.14 distinguished ATTR patients from AL patients with a 100% (CI: 72.25-100%) sensitivity and 100% (CI: 67,56-100%) specificity. Martineau et al. [27] observed a significantly higher TBR in ATTR amyloidosis patients (n=7) (0.98 ± 0.09) as compared to AL amyloidosis patients (0.85 ± 0.08; p = .026) and controls (0.82 ± 0.07; p = .020). A TBR cut-off of 0.89 resulted in a sensitivity of 75% (CI: 35-97%) and specificity of 100% (CI: 59-100%). No differences in mean SUV were found between ATTR amyloidosis patients, AL amyloidosis patients and controls. In the study by Morgenstern et al. [31], myocardial tracer uptake could visually be observed in ATTR amyloidosis patients (n=5), with a slightly higher uptake in ATTRwt amyloidosis patients compared to ATTRv amyloidosis patients, and no visual uptake in AL amyloidosis patients and controls. Mean SUV in ATTR amyloidosis patients (1.5 [1.4 - 1.7]) was significantly higher in comparison with controls (0.8 [0.4 - 0.9]; p = .012), but did not differ from the mean SUV in AL amyloidosis patients (0.95 [0.9 - 1.0]). In the study by Trivieri et al. [30] maximum TBR was significantly higher in ATTR amyloidosis patients (n=4) ( 1.14 ± 0.24) compared to AL amyloidosis patients (0.77 ± 0.06; p < .05) and controls (0.68 ± 0.04; p = .001). In the study by Zhang et al. [32], visual detection of ATTR amyloidosis on a PET scan 1 hour post injection had a sensitivity of 25% (CI: 8.9-53%) and a specificity of 100% (CI:57-100%). After 3 hours, sensitivity of visual detection was 30% (CI: 11-60%) and specificity remained 100%. A TBR cutoff of 0.76 distinguished ATTR-CM patients (n=12) from controls with 67% (CI: 39-86%) sensitivity and 80% (38-99%) specificity. TBR did not differ between ATTR-CM patients and controls after 1 and 3 hours in this study.

### Value of PET in follow-up of ATTR-CM patients

There were no studies found on the use of PET in the follow-up of patients with ATTR-CM for detection of treatment response or disease progression, with the exception of one case report on the use of serial [^18^F]FBB PET scanning in a single ATTRwt amyloidosis patient [19].

## Discussion

The primary aim of this review was to determine which PET-tracer has the highest accuracy to diagnose ATTR-CM. The reviewed studies evaluated the performance of five different tracers: [^11^C]PIB, Na[^18^F]F, [^18^F]FBP, [^18^F]FBB, and [^18^F]FMM. The results showed that [^11^C]PIB, Na[^18^F]F and [^18^F]FBP can be used to detect ATTR-CM, although it remains unclear whether [^18^F]FBP can be used to distinguish AL amyloidosis patients from ATTR amyloidosis patients. Furthermore, no studies on the use of PET to detect treatment response or disease progression in ATTR-CM patients were found.

The aim of this review was to determine which PET tracer has the highest accuracy to diagnose ATTR-CM but was complicated by a high heterogeneity in reported outcomes and small sample sizes leading to large insecurity (illustrated by broad confidence intervals) for accuracy. Accuracy was reported in only eight out of twenty-one included studies, investigating [11C]PIB (2 studies), [18F]FBB (2 studies) and Na[^18^F]F (4 studies). Although reported sensitivity for Na[^18^F]F was inferior to the other tracers in three out of four studies, Na[^18^F]F still seems a promising PET tracer. Inferior sensitivity can partially be explained by the selected study groups for analysis, ATTR amyloidosis versus AL amyloidosis for Na[^18^F]F and mainly CA versus controls for the other two tracers, as it might be easier to distinguish amyloidosis patients from controls compared with distinguishing subtypes of CA. Additionally, one of the Na[^18^F]F studies reported accuracy to distinguish between all included study groups (ATTR amyloidosis, AL amyloidosis and controls) by kinetic modelling instead of just two groups. Lastly, Andrews et al[28] reported a drastic improvement of sensitivity to 100% for Na[^18^F]F when uptake is linked to late gadolinium enhancement on cardiac magnetic resonance imaging, illustrating that method of (combined) image analysis is crucial for accurate diagnosis.

Furthermore, the majority of studies did not report accuracy outcomes. In order to take the findings of these studies into account when drawing a conclusion, differences in cardiac tracer uptake between study groups were also assessed. Based on this analysis it becomes clear that more research is needed, preferably in large prospective randomized cohorts established through international, multicentre collaborations. Evidence on differences between the various groups is limited and partially conflicting, especially for [^18^F]FBB and [^18^F]FMM. Differences in uptake of Na[^18^F]F are reported between ATTR amyloidosis patients and AL amyloidosis patients and controls. Also differences in [^11^C]PIB are reported between CA patients and controls and ATTR amyloidosis patients and controls, but comparative results between ATTR amyloidosis patients and AL amyloidosis patients are conflicting. Differences in [^18^F]FBP uptake have been described between CA patients and controls, but results from analysis of ATTR amyloidosis patients versus controls and AL amyloidosis patients are conflicting.

Our findings are only partially in line with those of a previous systematic review on the diagnostic accuracy of PET in cardiac amyloidosis by Kim *et al.* [8]*.* They found that [^11^C]PIB PET has a high diagnostic accuracy for detection of cardiac amyloidosis, while the sensitivity of Na[^18^F]F PET was low with a high specificity. Our systematic review includes 11 new studies in comparison to the systematic review of Kim et al., of which six investigated either [^11^C]PIB [13, 14, 16] or Na[^18^F]F [28, 29, 32], this probably led to different results and thus a different conclusion.

Currently, ATTR-CM is diagnosed either by bone scintigraphy, endomyocardial biopsy or a combination of an extracardiac biopsy and characteristic findings on CMR or echocardiography [4]. Bone scintigraphy yields high accuracy in diagnosing ATTR-CM patients, provided that AL amyloidosis has been ruled out by blood and urine tests [34]. In order for PET to be used as a non-invasive replacement for bone scintigraphy in the diagnosis of ATTR-CM, it would need a similar accuracy. Based on the results of this review, none of the investigated tracers have an accuracy comparable to the accuracy that is reported for bone scintigraphy [34]. However, PET may still be of added value in the care for ATTR-CM patients as it offers the possibility to quantify the amyloid load. Although recent efforts are made in finding methods to quantify tracer uptake on bone scintigraphy [35, 36], it remains challenging and is not yet common practice [37], unlike in PET. Quantification using kinetic modelling and retention index of PET tracer uptake in ATTR-CM patients could potentially allow for more accurate assessment of disease severity and prognosis, and particularly accurate monitoring of treatment response with the new generation medical drugs or for disease progression [21]. Furthermore, the binding mechanism of PET-tracers to amyloid deposits is different to the likely binding mechanism of bone scintigraphy tracers to amyloid deposits and could be more suitable for detecting subtle changes in amyloid load.

Four out of the five investigated tracers bind specifically to the beta-pleated motif of amyloid fibrils, regardless of the precursor protein [38]. In comparison to [^11^C]PIB, ^18^F-labelled tracers have some advantages, as these do not require an on-site cyclotron, have lower synthesis costs and a longer half-life. In contrast, Na[^18^F]F is thought to be incorporated into surface hydroxyapatite crystals through exchange of hydroxyl groups in active calcifications [39]. In ATTR amyloidosis, Na[^18^F]F is hypothesized to bind to microcalcifications in the vicinity of amyloid deposits and not to the amyloid fibrils themselves. [40, 41]. As Na[^18^F]F has a stronger affinity for ATTR amyloid deposits than for AL amyloid deposits [27–30], similar to the technetium-99m labelled bisphosphonates used in bone scintigraphy [34], it is a promising, easily clinically available tracer to distinguish between these types of CA [38].

In this review, we focused on investigating superiority of any of the PET tracers for the diagnosis of ATTR-CM in order to establish which tracers are most promising to use in the follow-up of these patients disease activity. Several studies noted that PET could possibly be suitable for detection of treatment response or disease progression in the follow-up of ATTR-CM patients, but did not provide any data to support this claim. Surprisingly, we only found one case report on the use of [^18^F]FBB in the follow-up of one ATTR-CM patient [19]. However, there is an increasing need for such studies, as multiple new, costly treatment options are currently implemented and new treatments are under investigation [7]. To facilitate personalized treatment optimization, clinicians could benefit from an imaging modality which accurately quantifies changes in amyloid load, such as PET.

One of the limitations of this systematic review is the lack of comparison of PET with conventional methods to diagnose ATTR amyloidosis patients, such as bone scintigraphy, cardiac magnetic resonance imaging or endomyocardial biopsy. Furthermore, a meta-analysis was not performed due to extremely high clinical heterogeneity resulting from variabilities in imaging protocols (different scanners, time intervals after tracer injection, acquisition times and tracer doses), outcome variables, composition of study groups, composition of control groups and cut-off values used to calculate accuracy. Although the problem of differing outcome variables and cut-off values could be overcome by obtaining the complete original data of each study, a meta-analysis would still lead to a non-sense pooled accuracy due to the differences between the study protocols and composition of the studied groups. Lastly, comparison of studies without clinical heterogeneity was not feasible, as selection of similarly conducted studies resulted in an extremely low number of included studies per tracer.

An important issue to further elaborate on, is the potential effect of the difference in time interval after tracer injection and different acquisition times on the results of the included studies, as suboptimal protocols could theoretically lead to false positive or false negative findings. Unfortunately, due to the different reported outcome variables, group compositions and tracer dosage use, a detailed comparison of all performed studies regarding the influence of start time and acquisition time for [^11^C]PIB is not feasible. However, when comparing a study with early and short image acquisition [14] (10 and 10 minutes respectively) with another study with late and longer image acquisition [13] (30 and 20 minutes respectively), the cut-off value of 1.09 resulted in similar sensitivity and specificity. No comparison of [^18^F]FBB was possible regarding the influence of acquisition times. Based on the dynamic [^18^F]FBP studies, in CA blood pool activity decreases early, whereas myocardial retention is present for around 60 minutes [22, 23]. In early and longer image acquisition (10 and 20 min respectively) SUV_mean_ is lower, compared to later and shorter image acquisition (40 and 10 min respectively), although these results could have also been a consequence of a lower tracer dose in the early and long image acquisition study (222MBq vs. 370MBq). In the two studies on [^18^F]FMM the tracer dose and the group composition of the disease of interest differs (CA vs. ATTR), but TBR_mean_ values were comparable between early and late acquisition for the amyloidosis and non-amyloidosis groups. Five of the six studies on Na[^18^F]F were performed using early scanning, at 60 minutes, one study scanned late at 60 minutes and 180 minutes. Due to lower TBR values of the 1 hour timepoint of the latter study, values were not comparable to the other five studies. Overall, it can be stated that a more generalized protocol and reporting of the outcomes across different studies would improve the head-to-head comparison of the effect of different duration after tracer injection and acquisition times considerably, since a definite conclusion on the effect on accuracy, sensitivity and specificity cannot be drawn at this time.

In conclusion, although heterogeneity of the included studies, [^11^C]PIB and Na[^18^F]F appear to be the most promising tracers for the detection of ATTR-CM and the distinction between ATTR and AL amyloidosis patients and/or controls. [^18^F]FBP can be used to detect CA, but might not be able to distinguish between different types of CA. Additional research to [^18^F]FBB and [^18^F]FMM is needed. The value of PET in follow-up of patients receiving disease specific treatment is lacking. Larger prospective studies to determine the diagnostic accuracy of PET to for ATTR are needed and future randomized PET studies are warranted in the follow-up of ATTR amyloidosis patients during therapy.

### Supplementary Information

ESM 1 (PDF 230 kb)

ESM 2 (PDF 199 kb)

ESM 3 (PDF 196 kb)

ESM 4 (DOCX 45 kb)

## Data Availability

No new data were generated or analysed in support of this research.

## Abbreviations

AA: Serum amyloid A amyloidosis
AL: Immunoglobulin light chain amyloid
ATTR: Transthyretin amyloid
ATTRv: Hereditary transthyretin amyloid
ATTRwt: Wild type transthyretin amyloid
ATTR-CM: Transthyretin amyloid cardiomyopathy
[^11^C]PIB: Carbon-11 Pittsburgh Compound B
CA: Cardiac amyloidosis
CI: Confidence interval
[^18^F]FBB: Fluorine-18 florbetaben
[^18^F]FBP: Fluorine-18 Florbetapir
[^18^F]FMM: Fluorine-18 Flutemetamol
HC: Healthy controls
HCM: Hypertrophic cardiomyopathy
MBq: Megabecquerel
MBq/kg: Megabecquerel per kilogram bodyweight
MV: Molecular volume
Na[^18^F]F: Fluorine-18 Sodium Fluoride
PET: Positron emission tomography
RI: Retention index
ROI: Region of interest
SUV: Standardized uptake value
SUVR: Standardized uptake value ratio
TBR: Tissue-to-background ratio
QUADAS-2: Quality Assessment of Diagnostic Accuracy Studies-2
