# Correction to: Positron emission tomography in the diagnosis and follow-up of transthyretin amyloid cardiomyopathy patients: A systematic review

**DOI:** 10.1007/s00259-023-06408-9

**Published:** 2023-08-22

**Authors:** H. S. A. Tingen, A. Tubben, J. H. van ’t Oever, E. M. Pastoor, P. P. A. van Zon, H. L. A. Nienhuis, P. van der Meer, R. H J. A. Slart

**Affiliations:** 1 Department of Nuclear Medicine and Molecular Imaging, University Medical Center Groningen, Hanzeplein 1, 9713GZ, Groningen, The Netherlands; 2 Amyloidosis Centre of Expertise, University Medical Center Groningen, Hanzeplein 1, 9713GZ, Groningen, The Netherlands; 3 Department of Cardiology, University Medical Center Groningen, Hanzeplein 1, 9713GZ, Groningen, The Netherlands; 4 Department of Internal Medicine, University Medical Center Groningen, Hanzeplein 1, 9713GZ, Groningen, The Netherlands; 5 Biomedical Photonic Imaging Group, Faculty of Science and Technology, University of Twente, Enschede, The Netherlands

**Correction to: European Journal of Nuclear Medicine and Molecular Imaging**

10.1007/s00259-023-06381-3

The authors regret that the name of J. H. van ’t Oever was incorrectly presented as J. H. A. van ’t Oever in the original article.

The original article has been corrected.

The original article can be found at 10.1007/s00259-023-06381-3.

