# Supplementary material for: Positron emission tomography in the diagnosis and follow-up of transthyretin amyloid cardiomyopathy patients: A systematic review
Source: Eur J Nucl Med Mol Imaging. 2023 Aug 10;51(1):93–109. doi: 10.1007/s00259-023-06381-3 (PMC10684414; doi:10.1007/s00259-023-06381-3)
Supplement: Supplementary file 1 — (PDF 230 kb) [file 259_2023_6381_MOESM1_ESM.pdf]

Supplementary information to:

Title: Positron emission tomography in the diagnosis and follow-up of transthyretin amyloid cardiomyopathy patients: A systematic review

Journal: European Journal of Nuclear Medicine and Molecular Imaging

Authors: Tingen HSA, MD*, Tubben A, MD,*, van 't Oever JH, BSc, Pastoor EM, BSc, van Zon PPA, BSc, Nienhuis HLA, MD, PhD, van der Meer P MD, PhD, Slart RHJA, MD, PhD

Shared first author *

Details corresponding author:

Hendrea Tingen

Amyloidosis Centre of Expertise

University Medical Center Groningen

Hanzeplein 1

9713GZ Groningen

h.s.a.tingen@umcg.nl

Online resource 1: Full search strategy Medline, Embase, the Cochrane library and Web Of Science

Electronic database	Search terms related to study population	Boolean operator	Search terms related to exposure
Medline	("Amyloidosis"[Mesh] OR ATTR [tiab] OR transthyretin [tiab] OR "cardiac amyloidosis" [tiab]) NOT “alzheimer” [ti] NOT “dementi*” [ti]	AND	("Positron-Emission Tomography"[Mesh] OR Positron-Emission Tomograph*[tiab] OR PET/CT[tiab] OR PET[tiab])
Embase	('amyloidosis'/exp OR attr:ab,ti OR transthyretin:ab,ti OR 'cardiac amyloidosis':ab,ti) NOT alzheimer NOT dementi*	AND	'positron emission tomography'/exp OR 'positron-emission tomograph*':ab,ti OR 'pet/ct':ab,ti OR pet:ab,ti
The Cochrane Library	MeSH descriptor: [Amyloidosis] explode all trees OR (attr):ti,ab,kw OR (cardiac amyloidosis):ti,ab,kw OR (transthyretin):ti,ab,kw NOT (alzheimer):ti NOT (dementi*):ti	AND	MeSH descriptor: [Positron-Emission Tomography] explode all trees OR (Positron-Emission Tomograph*):ti,ab,kw OR ("PET/CT"):ti,ab,kw OR (PET):ti,ab,kw
Web of Science	((((((((TI=(Amyloidosis)) OR AB=(Amyloidosis)) OR TI=(ATTR)) OR AB=(ATTR)) OR AB=(transthyretin)) OR TI=(transthyretin)) OR TI=(cardiac amyloidosis)) OR AB=(cardiac amyloidosis)) NOT TI=(alzheimer)) NOT TI=(dementi*)	AND	(((((((((TI=(Positron-Emission Tomography)) OR AB=(Positron-Emission Tomography)) OR AB=(Positron-Emission Tomograph*)) OR TI=(Positron-Emission Tomograph*)) OR TI=(PET/CT)) OR AB=(PET/CT)) OR TI=(PET)) OR AB=(PET))
