# Supplementary material for: Positron emission tomography in the diagnosis and follow-up of transthyretin amyloid cardiomyopathy patients: A systematic review
Source: Eur J Nucl Med Mol Imaging. 2023 Aug 10;51(1):93–109. doi: 10.1007/s00259-023-06381-3 (PMC10684414; doi:10.1007/s00259-023-06381-3)
Supplement: Supplementary file 2 — (PDF 199 kb) [file 259_2023_6381_MOESM2_ESM.pdf]

Supplementary information to:

Title: Positron emission tomography in the diagnosis and follow-up of transthyretin amyloid cardiomyopathy patients: A systematic review

Journal: European Journal of Nuclear Medicine and Molecular Imaging

Authors: Tingen HSA, MD*, Tubben A, MD,*, van 't Oever JH, BSc, Pastoor EM, BSc, van Zon PPA, BSc, Nienhuis HLA, MD, PhD, van der Meer P MD, PhD, Slart RHJA, MD, PhD

Shared first author *

Details corresponding author:

Hendrea Tingen

Amyloidosis Centre of Expertise

University Medical Center Groningen

Hanzeplein 1

9713GZ Groningen

h.s.a.tingen@umcg.nl

Online resource 2: List of included items in the data extraction form

Category	Parameter
Study characteristics	First author
	Title
	Year
	Journal
	Country
	DOI
	Funding source
Imaging protocol	Type of PET scanner
	Tracer
	Tracer dose
	Acquisition times
	Placement of ROI
Characteristics study and control population	Condition
	Number of patients
	Age
	Percentage male
	Ethnicity
	Mode of diagnosis
	Organ involvement (for ATTR amyloidosis patients only)
	Functional class (for ATTR amyloidosis patients only)
	Treatment (for ATTR amyloidosis patients only)
	Medical history
	Co-medication
	Presence of cardiac implantable devices
Laboratory tests of study group and control population	NT-proBNP
	BNP
	Troponin T
	Troponin I
	Haemoglobin
	Calcium

	Kappa/Lambda free light chains + ratio
	Creatinine
	Albumin
	eGFR
Electrocardiogram	Percentage low voltage
	QRS time
	Percentage cardiac hypertrophy
Echocardiogram	Intraventricular septal wall thickness
	Left ventricular ejection fraction
	Left ventricular mass
	Left ventricular mass index
	Left ventricular posterior wall thickness
	Left ventricular diastolic diameter
	Left ventricular end-diastolic volume index
	Left ventricular systolic diameter
	Left ventricular end-systolic volume index
	Left ventricular global longitudinal strain
	LA volume index
	Tricuspid annular plane systolic excursion
	E
	E/A ratio
	E'
	E/E'
	Right ventricular wall thickness
	Diastolic dysfunction grade
	Pulmonary artery systolic pressure
Diagnostic accuracy	Specificity visual interpretation
	Sensitivity visual interpretation
	Accuracy visual interpretation
	Specificity quantitative analysis
	Sensitivity quantitative analysis
	Accuracy quantitative analysis
	Cut-off quantitative analysis

	Positive predictive value quantitative analysis
	Negative predictive value quantitative analysis
	Area under the curve
	Receiver operating characteristic
	SUVmean
	SUVmax
	Target-to-background ratio
	Retention index
	p-values of all tests
Follow-up	Follow-up time
	Change in SUVmax
	Change in SUVmean
	Change in target-to-background ratio
	Change in retention index
	Comparison with conventional follow-up
