# Supplementary material for: Positron emission tomography in the diagnosis and follow-up of transthyretin amyloid cardiomyopathy patients: A systematic review
Source: Eur J Nucl Med Mol Imaging. 2023 Aug 10;51(1):93–109. doi: 10.1007/s00259-023-06381-3 (PMC10684414; doi:10.1007/s00259-023-06381-3)
Supplement: Supplementary file 3 — (PDF 196 kb) [file 259_2023_6381_MOESM3_ESM.pdf]

Supplementary information to:

Title: Positron emission tomography in the diagnosis and follow-up of transthyretin amyloid cardiomyopathy patients: A systematic review

Journal: European Journal of Nuclear Medicine and Molecular Imaging

Authors: Tingen HSA, MD*, Tubben A, MD,*, van ’t Oever JH, BSc, Pastoor EM, BSc, van Zon PPA, BSc, Nienhuis HLA, MD, PhD, van der Meer P MD, PhD, Slart RHJA, MD, PhD

Shared first author *

Details corresponding author:

Hendrea Tingen

Amyloidosis Centre of Expertise

University Medical Center Groningen

Hanzeplein 1

9713GZ Groningen

h.s.a.tingen@umcg.nl

Online resource 3: Assessment of overlap in study populations between studies

All studies were assessed for the risk of overlap in study populations by comparing characteristics of the studies, such as research group, location and time of inclusion, imaging protocol, composition of study group and control group and age and sex distribution of the study group. The risk was assessed per subgroup of studies investigating the same tracer.

In the six studies investigating [^{11}C]PIB, two [16], [17] and three [12], [14], [15] studies were conducted by the same research group or resulted from a collaboration. Of the three studies conducted by the Swedish research group / collaboration, the first study was a retrospective study [12] and the last two studies were prospective studies [14], [15]. Imaging protocols varied between the different studies, scanning at different moments after tracer injection, for different durations and with a different administered tracer dose between the studies. Furthermore, two studies investigated ATTR amyloidosis patients only [14], [15], while the third study investigated both AL and ATTR amyloidosis patients [12]. All studies compared amyloidosis patients with healthy controls, but one study also included patients with hypertrophic cardiomyopathy in the control group [14]. Based mainly on the differing imaging protocols, the risk of overlap in study populations was deemed low.

The two studies from the Japanese research group [16], [17] used identical imaging protocols. Both studies are prospective and include ATTR amyloidosis patients only. Both studies published and overview table of the characteristics of all included patients, and these were compared. Four patients were identified to possibly be included in both studies based on the mutation, age, sex, presence of cardiac amyloidosis and presence of cardiac [^{18}F]PIB uptake on PET. Although both studies investigate a different method of analysis and therefore contribute to a better understanding of the potential of [11C]PIB PET in ATTR-CM, inclusion of patients in both studies could have an impact on the results, especially in one of the studies,

where the total study group consisted of seven ATTR-CM patients [17]. Therefore, the risk of overlap between these two studies was scored as high.

Two studies to the use of [^{18}F]FBB PET were conducted by the same study group [18], [21]. The firstly conducted study was prospective and investigated the use of dynamic and static PET [18], while the second study was retrospective and investigated the use of dynamic PET and kinetic modelling [21]. Although dynamic imaging protocols differ slightly, with a scan duration of 60 minutes in the first study [18] and 40 or 45 minutes in the second study [21], protocols are still so similar that overlap of study population could be possible. Both studies include AL and ATTR amyloidosis patients and include hypertrophic cardiomyopathy and hypertensive heart disease patients as control group, although one of the studies also includes patients with dilated cardiomyopathy in the control group [18]. As details of the inclusion process (e.g. timing and location) are not reported in the retrospective study [21], and as this study includes less patients that the prospective study, the risk of overlap was deemed unclear.

Of the studies to the use of [^{18}F]FBP and [^{18}F]FMM, none of the studies were conducted by the same research group. The risk of overlap was therefore scored low.

Two sets of studies investigating the diagnostic accuracy of Na[^{18}F]F were conducted by the same research group or collaboration [27], [28], [30], [32]. The studies conducted by the Scottish / American collaboration [28], [30], used different imaging protocols. Both studies included both AL and ATTR amyloidosis patients and compared the outcomes of these patients with those of healthy controls. One of the studies also included aortic valve stenosis patients [28]. Both studies are prospective, although the details of the inclusion process are not clear for the study by Trivieri et al. [30]. However, as inclusion of patients for Andrews et al. [28] started in December 2015 and the study by Trivieri et al. [30] was

published in December 2016, overlap of inclusion periods could have occurred. However, due to the highly varying imaging protocols, the risk of overlap was considered to be low.

The studies published by the Canadian study group [27], [32] used equal dynamic imaging protocols. One of the studies was prospective and included patients from January 2018 until July 2020 [32], the other study was retrospective and included patients scanned between October 2017 and September 2018 [27], resulting in an overlapping period of inclusion of nine months. Although the prospective study included ATTR amyloidosis patients only, and the retrospective study included both AL and ATTR amyloidosis patients, and the control groups differed between the studies, there is still a possibility of ATTR amyloidosis patients being included in both studies. Therefore the risk of overlap was deemed unclear.
