# Supplementary material for: Positron emission tomography in the diagnosis and follow-up of transthyretin amyloid cardiomyopathy patients: A systematic review
Source: Eur J Nucl Med Mol Imaging. 2023 Aug 10;51(1):93–109. doi: 10.1007/s00259-023-06381-3 (PMC10684414; doi:10.1007/s00259-023-06381-3)
Supplement: Supplementary file 4 — (DOCX 45 kb) [file 259_2023_6381_MOESM4_ESM.docx]

**Supplementary information to:**

***Title:*** Positron emission tomography in the diagnosis and follow-up of transthyretin amyloid cardiomyopathy patients: A systematic review

***Journal:*** European Journal of Nuclear Medicine and Molecular Imaging

***Authors***: Tingen HSA, MD^*^, Tubben A, MD^,*^, van ’t Oever JH, BSc, Pastoor EM, BSc, van Zon PPA, BSc, Nienhuis HLA, MD, PhD, van der Meer P MD, PhD, Slart RHJA, MD, PhD
*Shared first author ^*^*

***Details corresponding author:***Hendrea Tingen
Amyloidosis Centre of Expertise
University Medical Center Groningen
Hanzeplein 1
9713GZ Groningen
[h.s.a.tingen@umcg.nl](mailto:h.s.a.tingen@umcg.nl)

**Online resource 4: Reference standard used in included studies**

| Authors, year (reference no.) | Short description reference standard | Additional information |
| --- | --- | --- |
| Antoni,  2012 [12] | Clinical diagnosis based on:   - Immunohistochemistry (subcutaneous tissue + myocardial tissue) - Echocardiography (Gertz et al) |  |
| Bi,  2022 [13] | Clinical diagnosis based on:   - Histopathological analysis and immunohistochemistry (subcutaneous tissue + bone marrow) - Echocardiography - CMR | Cardiac involvement: Thickening of septal wall on echocardiography plus any two of the following:  a) granular, speckled or ground glass echo  b) unexplained low voltage <0.5 mV on ECG  c) decreased left ventricular diastolic function  d) enlarged left atrium  or LGE on CMR |
| Rosengren,  2020 [14] | Clinical diagnosis based on:   - Histopathological analysis (endomyocardial and abdominal fat) - Echocardiography (Gertz et al) - CMR |  |
| Pilebro,  2018 [15] | Clinical diagnosis based on:   - Genetic testing - Histopathological analysis and immunohistochemistry (fat pad) - Blood tests - Echocardiography | Blood tests: Trop T en NT-proBNP |
| Takasone,  2020 [16] | Clinical diagnosis based on:   - Genetic testing - Histopathological analysis and immunohistochemistry - Blood and urine tests - Echocardiography - Electrocardiography - BNP | Cardiac involvement:  Echocardiography: increased left ventricular wall thickness >12 mm  Electrocardiography: combination of low voltage in limb lead and QS pattern in anterior precordial lead, sick sinus syndrome, AV-block and/or atrial fibrillation  BNP > 100 pg/mL |
| Ezawa,  2018 [17] | Clinical diagnosis based on:   - Histopathological analysis and immunohistochemistry - Genetic testing - Echocardiography (Gertz) |  |
| Genovesi,  2021 [18] | Clinical diagnosis based on:   - Blood and urine tests - Genetic testing - Electrocardiography - Doppler echocardiography - Bone scintigraphy - CMR - Histopathological analysis | Blood tests: NT-proBNP, hsTrop T, urine and/or serum immunoglobulin light chains, plasma protein electrophoresis and serum free light chains |
| Kircher,  2019 [19] | Clinical diagnosis based on:   - Histopathological analysis - Blood tests - Echocardiography - Bone scintigraphy - CMR | Blood tests: NT-proBNP and cTrop T |
| Law,  2016 [20] | Clinical diagnosis based on:   - Histopathological analysis (endomyocardial tissue, kidney, bone marrow, prostate, rectum) - Subtyping by mass spectrometry, immunohistochemistry or immunofluorescence - Geneteic testing - Blood tests - Echocardiography (Gertz) | Cardiac involvement: consensus criteria of the international society of amyloidosis for cardiac involvement.  Blood tests: Trop I en BNP |
| Santarelli, 2022 [21] | Clinical diagnosis based on:   - Histopathological analysis - Blood and urine tests - Electrocardiography - Echocardiography - Bone scintigraphy - CMR | Blood and urine tests: NT-proBNP, hs Trop T, IG light chains in serum and/or urine |
| Dorbala,  2014 [22] | Clinical diagnosis based on:   - Histopathological analysis, immunohistochemistry and mass spectrometry (endomyocardial tissue, extracardiac tissue) - Echocardiography | Cardiac involvement: wall thickness measurements of >11 mm, bright echogenic myocardium  Echocardiography: Mitral inflow parameters, pulmonary venous doppler information or tissue doppler imaging at mitral septal and lateral annulus |
| Osborne,  2015 [23] | Clinical diagnosis based on:   - Histopathological analysis (endomyocardial tissue) - CMR |  |
| Mestre-Torres,  2018 [24] | Clinical diagnosis based on:   - Histopathological analysis - Blood and urine tests - CMR - Echocardiography | Blood and urine tests: hsTrop T, BNP, urea, creatinine, eGFR, proteinuria |
| Dietemann,  2019 [25] | Clinical diagnosis based on:   - Histopathological analysis (extracardiac tissue) - Echocardiography - Bone scintigraphy - CMR | Echocardiography: cardiac morphology and echogenicity of the myocardium  CMR: Modified look-locker inversion recovery for high resolution T1 mapping and LGE |
| Papathanasiou,  2020 [26] | Clinical diagnosis based on:   - Histopathological analysis (myocardial) - Blood and urine tests - Bone scintigraphy - Electrocardiography - Echocardiography - CMR | Blood and urine tests: serum free light chain assay, serum and urine immunofixation and serum protein electrophoresis |
| Martineau,  2019 [27] | Clinical diagnosis based on:   - Histopathological analysis - Bone scintigraphy - Blood tests | Blood tests: serum protein electrophoresis |
| Andrews,  2020 [28] | Clinical diagnosis based on:   - Histopathological analysis - CMR |  |
| Abulizi,  2019 [29] | Clinical diagnosis based on:   - Histopathological analysis and immunohistochemistry (endomyocardial tissue, extracardiac tissue) - Electrocardiography - Echocardiography - Blood tests | Blood tests: serum assays of ultrasensitive cardiac Trop T, NT-proBNP, creatinine |
| Trivieri,  2016 [30] | Clinical diagnosis based on:   - Histopathological analysis |  |
| Morgenstern, 2017 [31] | Clinical diagnosis |  |
| Zhang,  2020 [32] | Clinical diagnosis based on:   - Histopathological analysis - Bone scintigraphy - Blood tests | Blood tests: serum protein electrophoresis with immunofixation and serum free light chain assay |
| *Trop T, Troponin T; Trop I, Troponin I; hs, high sensitive; c, cardiac; BNP, brain natriuretic peptide; NT-proBNP, N-terminal pro brain natriuretic peptide; IG, immunoglobulin; ECG, electrocardiogram; LGE, late gadolinium enhancement; CMR, cardiac magnetic resonance imaging.* | | |
